# Supplementary material for: “It’s like a book in the palm of my hand”: Adapting the Safe Delivery App for Papua New Guinea to improve quality of maternal and newborn care
Source: PLoS One. 2025 Aug 8;20(8):e0324102. doi: 10.1371/journal.pone.0324102 (PMC12334014; doi:10.1371/journal.pone.0324102)
Supplement: S1 File — (DOCX) [file pone.0324102.s001.docx]

Contents

[Acceptability 1](#_Toc202437280)

[Supporting clinical care 2](#_Toc202437281)

[Transfer of knowledge and information 2](#_Toc202437282)

[Using the App to support and test knowledge 3](#_Toc202437283)

[Key aspects and modules used 4](#_Toc202437284)

[Challenges 4](#_Toc202437285)

[Strategies to support the use of the App 4](#_Toc202437286)

[Recommendations 4](#_Toc202437287)

[Alignment with National Guidelines 5](#_Toc202437288)

[Endorsement from National Societies 5](#_Toc202437289)

# Acceptability

Video clips

Action cards

I’m working up in Enga Province, which is up in the Highlands. So it’s in rural… this app it’s really helpful. What I see is like most of us working in rural, sometimes we work without doctors and HEOs and we [as a ]midwife work alone. So as a student midwife; I worked as a nurse and [/but/] as a student midwife, I see that the app is like it really helps us. So I wish to go back and share this with some of my colleague midwives in hospital. Educator

Maria, Student midwife, Enga Province (FGD)

I wish I have this application long time ago.

This is very handy; it’s like a book on my palm I just carrying it anywhere.

Susan, Midwife Educator, East Sepik Province (FGD)

Well, I will say all of them are important. All of them are user-friendly. Because coming from an education background, we have different types of learners… there is a learner that will have to do something to follow, there is a learner that by just seeing it, just watching the video will already understand, there is another person that has to read and understand. So up on that, the whole app itself is so useful. So you are serving the different types of people that you are sharing this app to. So no one is missing in the gap. So that’s how I see it from an education background.

Hilda, Midwife Educator, Eastern Highlands Province

Maybe recently we the Morobe we took one batch through on this delivery app when taking EmONC training as well with my bosses. They were running the workshop and they gave us this App. And like me, I have a network out at the rural communities that they used to call me up if they are facing any complications. So I simply asked them if they have an android phone and then I instructed them to install it. And then the feedback they gave me was it was overwhelming. Like they got the updated information and it’s very helpful. And this app already saved one mother with retain placenta; with the community health worker, it’s not a midwife that performed. So I think it is very helpful. Yeah.

Lily, Midwife, Office in Charge, Morobe Province (FGD)

# Supporting clinical care

Usage

Key modules referred to

**“**According to my statistics (data), I don’t do manual removal of placenta now. Not at all, because of the delivery app and the active management of first stage of labour. Especially the bladder, especially the bladder (emptying the bladder with a urinary catheter) and the repeat of oxytocins…those are the two things my staff have learnt in active management of first stage of labour and then they are putting into practice and that brings change in their performance...So we [they] no longer come up [to the] centre now and I don’t do the manual removal of placenta because they follow the protocol they have seen in the delivery app”.

*Jackie, Senior Midwife and Unit Manager, Bougainville*

*According to my assessment, my staff can do appropriate hypertension and management of PPH. They can be able to fully manage PPH, that’s including my HEO…in the unit, she improved a lot.*

Jackie, Senior Midwife and Unit Manager, Bougainville

### Transfer of knowledge and information

“… the training that was given to us was for all the midwifery schools in the country, so as trainers in the institutions we got our knowledge so we went back and then imparted the knowledge [to] our midwives. So when the midwives then go to the facility, they will have to train their own staff in the facility. So this is transfer of information. So we don’t need to get someone to do more training, it’s going to be cost[ly]. So we just transfer the information. And only that, then the health care workers then follow the instructions and save the mothers.

Hilda, Midwife Educator, Eastern Highlands Province

[its] an app for all, not just for midwives…not only for midwives but I would say we could share that knowledge with all the health workers.

Hilda, Midwife Educator, Eastern Highlands Province

…so students are able to differentiate the hypertension, preeclampsia. They are able to uh differentiate severe preeclampsia and eclampsia, and at least they know how to treat them. Because the directives are also there, how they can use the magnesium sulphate and all these.

Philippa, Midwifery Lecturer, National Capital District, Midwifery Lecturer, National Capital District

*So I’ve followed up with them [two previous cohorts of student midwives] and they are using it so it’s a—they say it is very helpful…And this year, uh yeah just last month we had our—completed our EmONC training as well. So on the last day, I gave them the safe delivery app as well so they are using it. They are currently using it, even doing their studies for exams.*

Philippa, Midwifery Lecturer, National Capital District, Midwifery Lecturer, National Capital District

Magnesium sulphate…Sometimes it is the community health workers who are on shift and they come across mothers with preeclampsia so they just let me know that they have come across a case so they wanted to give magnesium sulphate. So I just tell them to—I usually tell them to go ahead and give it. Before that, they need supervision like a HEO or a midwife is to stay with them before they give magnesium sulphate. Now they give it on their own.

Jackie, Senior Midwife and Unit Manager, Autonomous Region of Bougainville (FGD)

I’m working in the hospital, urban hospital and currently (*clear throat*), currently I am using this app. Uh they were doing a presentation about the newborn resuscitation and some of the practice they did was a bit outdated, so I showed them this app, how to do newborn resuscitation. So, when they go through that app, they saw that yes the information is updated. The practice that they did was not accordingly so when they go through this newborn resuscitation procedure, they could pick out the updated management.

Raylyn, Midwife, Bouganvile (FGD)

In Papua New Guinea, we don’t have midwives in all the health centres and aid-posts. Most of the midwives are either in the hospital, tertiary hospitals, or in the companies in the health centres you would only have one midwife. For health centres and aid-posts, zero. But if you can have this information, this app onto a phone and the phone must be in the health facility, not for the OIC to take it around because that’s their personal phone they can go anywhere with it, but anything that belongs to the facility it remains within the facility, and then if a CHW is attending to a labouring mum and there is a complication, just click on the phone and the app is there so go and follow all the instructions. So for a midwife to have it, being useful [/using/] the app, she has to train her staff within the facility after the graduation. So that we share the knowledge and skills with other health care workers within the facility where they will help save the lives of the mothers during the absence of a midwife.

Hilda, Midwife Educator, Eastern Highlands Province, Midwife Educator, Eastern Highlands Province

### Using the App to support and test knowledge

But I encourage my students to at least we can all go through the my learning just to test our wealth of knowledge.

Philippa, Midwifery Lecturer, National Capital District, Midwifery Lecturer, National Capital District

**…**from the midwifery knowledge that we gained, we can, we feel, yeah like it was a tool use to like confirm or cross-check what I—the knowledge I’ve learnt in school, and then like recheck and referring to them...I have my, the red obstetrics and gynaecology book with me so it’s like I have those two tools. But I don’t, I work—in a week maybe once, maybe once or twice in a week, not really. I don’t normally use it every day but if I wouldn’t have that red book with me then I would have depended more on the app.

Mary Research Midwife, Madang Province

I did find it very helpful, especially in this study when we were coming across many mothers who were being identified as pre-eclamptic mothers or hypertensive mothers. Though we had the red obstetric gynecology handbook but some of the information which were in the app were, were really helpful when considering in managing cases of this—of the mothers who were—who had pre-eclampsia or high blood pressure.

Ben, Research Midwife, East New Britain

### Key aspects and modules used

…retain placenta, uh neonatal resuscitation, post-partum haemorrhage, also the hypertension, especially the severe preeclampsia, uh and also the active third stage management, and normal labour, the maternal sepsis, uh neonatal management. So these are the most topics that I emphasise

Philippa, Midwifery Lecturer, National Capital District, Midwifery Lecturer, National Capital District

So after talking with her and her husband, we brought her to the health centre… there wasn’t any like senior midwife there, just me and [another nurse] and she wasn’t experienced in giving magnesium sulphate as she was a junior CHW at that time. So, I was like okay, this is what we will do. So I got out the app, I was watching it the whole time because I knew about the things of hypertension. So I was like, okay we will do this and this. So that was what we did - we followed the procedures and I got [the nurse] to help me out. So she is going to go for her training next year for midwifery. But I didn’t like actually introduce her to the app, I just like involved her with the procedures. So that’s like one experience I had with the app.

Mary Research Midwife, Madang Province

*… I’d say the language used in the videos, some of the terms that are used, um they are—they are clear. But the ones that are written you know, down in the action cards and coming down, the ones that are in writing, let’s say I think some of the terms in there would be a bit—uh I’d say would be a bit hard to understand unless they have you know, their medical dictionary there they can easily refer to the terms, what’s its meaning or referring to.*

Ben, Research Midwife, East New Britain

# Challenges

### Strategies to support the use of the App

what I want to recommend here is that we need to have more training in the region. More training especially for the health workers working in the rural health facilities, and especially the midwives or those who are actually working in the labour ward. Like, there would [/must/] be more trainings.

Jackie, Senior Midwife and Unit Manager, Bougainville

Currently, almost, let me say 70, 80 percent of the population would have access to a cell phone and especially an android so they are able to download the app so they are able to access it when they needed it. But the other approach; that’s for individual midwives, but if we could advice them to purchase a phone for the facility so when a midwife travels out, she goes with the phone but the other phone is for the facility will have the app already there and it will be left in the health centre and any person attending to a labouring mum or antenatal mum would always have access to the phone at the facility. That is another approach we could have.

Hilda, Midwife Educator, Eastern Highlands Province

# Recommendations

…let’s say hypertension management, like checking the air way and breathing, maybe how they positioned [the woman]. Especially the women, because they—because women are pregnant and the way we treat them and put them to the side, maybe just the image how we can place them [lying on their left side]

Philippa, Midwifery Lecturer, National Capital District, Midwifery Lecturer, National Capital District

I’d like to add on something, just one thing, when I was going through the app I realise that some of the IMOrg complication, I understand that in Australia you don’t do vacuum and breech and twins, and I think it would be lovely too if you add these ones. Like the steps in doing twin delivery, um vacuum, breech presentation and other IMOrg procedures. That will be helpful for us the remote midwives.

Val, Student midwife, Autonomous Region of Bougainville (FGD)

And I prefer if we can also have it translated to the pidgin (Tok Pisin) language, so yeah it can be most easy for those that who are in the remote area who are trying to help us.

Michelle, Midwife, Central Province (FGD)

### Alignment with National Guidelines

…because we are introducing it into Papua New Guinea and we need to go in line with the current practice that we have. But we are happy for whatever is evidence based or is current, that can be added on. Maybe we have to update our red book. So if we are behind scheduled then with the app, with the current evidence based, I’m happy for it. As long as it is evidence based information then we can say, “Okay, PNG Medical Society or Obstetric Gynaecology Society maybe we need to review our red book or standard manual so that we go in line with the current practice.”

Hilda, Midwife Educator, Eastern Highlands Province

*Oh yes, it was straight forward because most of the contents that were in the app were similar to the EmONC training that was previously run, I think it was by the—one of the organizations they were doing training on emergency and obstetric care. So most of the contents that were in the application were similar to the courses they were taught in that short course. So when I happen to download and open up the app, I could easily refer to—back to the training which was given.*

Ben, Research Midwife, East New Britain

### Endorsement from National Societies

So it has to go there to be approved so that in the event that there is a legal implication, then we are safeguarded; you and me.

Hilda, Midwife Educator, Eastern Highlands Province
